# Trends in Suicide in Japan Following the 2019 Coronavirus Pandemic

**DOI:** 10.1001/jamanetworkopen.2022.4739

**Published:** 2022-03-29

**Authors:** Nobuyuki Horita, Sho Moriguchi

**Affiliations:** 1 Chemotherapy Center, Yokohama City University Hospital, Yokohama, Japan; 2 Department of Neuropsychiatry, Keio University School of Medicine, Tokyo, Japan

## Abstract

This cohort study examines trends in suicide rates in Japan from 2009 to 2021, including the COVID-19 pandemic.

## Introduction

During the coronavirus disease 2019 (COVID-19) pandemic, there were concerns that suicides would increase due to changes in lifestyle that restricted human contact in schooling, employment, and social activities. Although several studies reported little or no increase in suicide rates in the early phase of the pandemic,^1,2,3,4^ to our knowledge there is no detailed report for the entire first year of the pandemic or longer. This study analyzed suicide data in Japan, where crude suicide incidence ranked fourth among 33 Organization for Economic Cooperation and Development countries.

## Methods

This retrospective cohort study analyzed Japanese Ministry of Health, Labour and Welfare data on the monthly number of individuals who died of suicide for all Japanese residents between January 2009 and September 2021.^4^ Data from the ministry had reasonable consistency with data derived from other sources, such as National Police Agency reports. This study followed the Strengthening the Reporting of Observational Studies in Epidemiology (STROBE) reporting guideline for cohort studies. Review by the Japanese Ministry of Education, Culture, Sports, Science and Technology was not required, and informed consent was not necessary, because this study used publicly available deidentified data.

To estimate the monthly mortality incidence per 100 000 population, a multiple regression analysis was performed with the year, month, and a dummy variable for the period from April 2020 to September 2021 (referred to as the COVID-19 pandemic) as independent variables. The estimated incidence without the pandemic was calculated by inputting null for the dummy variable into the formula derived from the multiple regression analysis. Statistical analysis was conducted in BellCurve for Excel version 3.21 (Social Survey Research Information Co). The significance threshold was set at *P* < .05 in 2-sided tests.

## Results

The population size of Japan gradually decreased from 128 million to 126 million between 2009 and 2021, while the average age increased from 43 to 47 years. The annual suicide rate per 100 000 population was 20.9 for men and 8.7 for women in the fiscal year 2019 (April 2019 to March 2020), the period just before the pandemic.

**Figure 1.**
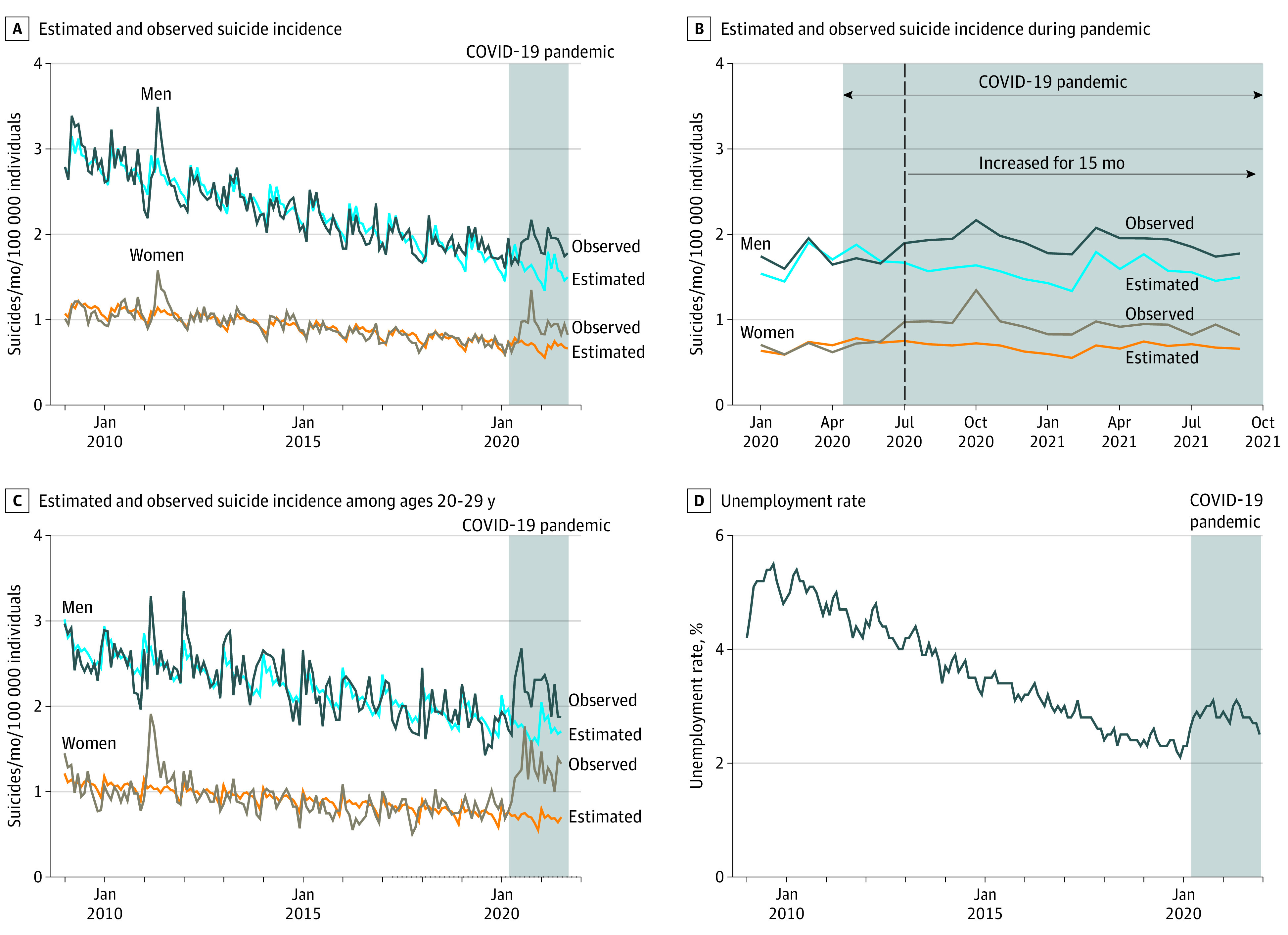
Suicide Incidence Throughout the COVID-19 Pandemic Suicide surged temporarily in 2011 following the Great East Japan Earthquake.

**Figure 2.**
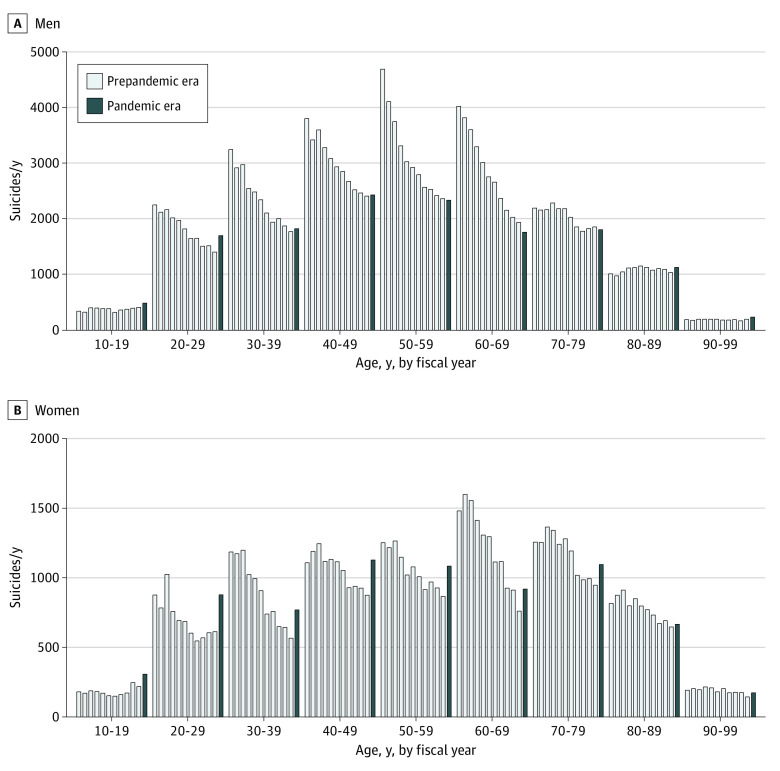
Sex- and Age-Stratified Suicide Case Numbers, 2009 to 2021 Annual data is based on the fiscal year, which in Japan starts in April. For example, the fiscal year 2020 (in this figure, the start of the pandemic era) means the term from April 2020 to March 2021.

In the gender-stratified analysis of all ages, the incidence of suicide was higher than the estimation by 17.0% (95% CI, 11.4%-22.7%; *P* < .001) for men and 31.0% (95% CI, 22.8%-39.2%; *P* < .001) for women (Figure 1A). Compared with the estimation, the monthly suicide incidence shifted upward after July 2020 following no significant increase during the early phase of the pandemic, April to June 2020 (women, 0.92 deaths per 100 000 population in April 2021 vs 0.62 deaths per 100 000 population in April 2020; men, 1.96 vs 1.65 deaths per 100 000 population) (Figure 1B). Clear surges of suicide incidence among women and men in their 20s were noted (women, 1.10 deaths per 100 000 population in April 2021 vs 0.69 deaths per 100 000 population in April 2020; men, 2.34 vs 1.88 deaths per 100 000 population) (Figure 1C, Figure 2). Suicide incidence showed a similar trend with the unemployment rate since 2009 (Figure 1D). Seasonal variation did not significantly affect these trends (Figure 1B).

## Discussion

Our data revealed an increase in the number of suicides especially in younger women, regardless of the season. The results are not in complete agreement with previous reports,^1,2,3,4^ for example, our data denied seasonable variability. The lack of increase of suicide in the early phase of the pandemic might be explained by some protective effect. We observed an association between suicide incidence and the unemployment rate (Figure 1A, Figure 1D).^5^ A 2021 study conducted in the US^1^ revealed a decline in suicide among White residents and increased suicide among Black residents. The association of COVID-19 with suicide may be different depending on race and sociodemographic factors. Results similar to our study were found in Germany. Benke et al^6^ reported that stay-at-home orders were associated with greater anxiety and loneliness, and younger adults were an especially vulnerable group for depression and anxiety due to restrictions on social behaviors.

We only analyzed homogenous data from Japan; thus, external validity for other countries was not validated. Inability to demonstrate causality due to the pandemic, unemployment rate, and welfare policies was another limitation to this study.

This cohort study examining national suicide data in Japan through September 2021 found that the COVID-19 pandemic was associated with an increase in suicide overall, and a specific increase among younger women. Our data highlighted a suicide increase that lasted more than a year in Japan after the initial phase of the pandemic.
